# Multi-scale integration and predictability in resting state brain activity

**DOI:** 10.3389/fninf.2014.00066

**Published:** 2014-07-24

**Authors:** Artemy Kolchinsky, Martijn P. van den Heuvel, Alessandra Griffa, Patric Hagmann, Luis M. Rocha, Olaf Sporns, Joaquín Goñi

**Affiliations:** ^1^Department of Informatics, School of Informatics and Computing, Indiana UniversityBloomington, IN, USA; ^2^Instituto Gulbenkian de CiênciaOeiras, Portugal; ^3^Department of Psychiatry, Rudolf Magnus Institute of Neuroscience, University Medical Center UtrechtUtrecht, Netherlands; ^4^Signal Processing Laboratory 5, Ecole Polytechnique Fédérale de LausanneLausanne, Switzerland; ^5^Department of Radiology, Lausanne University Hospital (CHUV) and University of Lausanne (UNIL)Lausanne, Switzerland; ^6^Department of Psychological and Brain Sciences, Indiana UniversityBloomington, IN, USA

**Keywords:** human connectome, resting-state, integrative regions, information theory, multivariate mutual information, complexity measures

## Abstract

The human brain displays heterogeneous organization in both structure and function. Here we develop a method to characterize brain regions and networks in terms of information-theoretic measures. We look at how these measures scale when larger spatial regions as well as larger connectome sub-networks are considered. This framework is applied to human brain fMRI recordings of resting-state activity and DSI-inferred structural connectivity. We find that strong functional coupling across large spatial distances distinguishes functional hubs from unimodal low-level areas, and that this long-range functional coupling correlates with structural long-range efficiency on the connectome. We also find a set of connectome regions that are both internally integrated and coupled to the rest of the brain, and which resemble previously reported resting-state networks. Finally, we argue that information-theoretic measures are useful for characterizing the functional organization of the brain at multiple scales.

## Introduction

The human brain is characterized by complex functional and structural organization at different scales. Both structural and functional aspects of large-scale brain organization can be studied using magnetic resonance imaging (MRI) technology. On the one hand, functional activity can be estimated from the blood-oxygen-level dependent (BOLD) signal recorded by functional MRI (fMRI) of gray matter. The pattern of correlations between the BOLD activities of pairs of regions determines the *functional connectivity*. On the other hand, the *structural connectivity*, or network of anatomical connections between brain regions also called the *human connectome* (Sporns et al., 2005; Sporns, 2013), can be inferred from the orientation of constrained diffusion throughout the brain as measured by diffusion spectrum imaging (DSI).

Recent research has sought to characterize the different functional and structural properties of different brain regions, both in “bottom-up” terms, by assigning distinct roles to localized regions, as well as in “top-down” terms, by decomposing the entire brain into interpretable networks or subsystems. For example, many functional studies have investigated *functional hubs*, or regions that maintain strong correlations with many other regions (Achard et al., 2006; van den Heuvel and Sporns, 2013). Other studies have decomposed resting-state time series into maximally independent components, where regions within the same components display correlated patterns of activation (Beckmann et al., 2005; Damoiseaux et al., 2006; Fox and Raichle, 2007; Smith et al., 2009; Yeo et al., 2011; Moussa et al., 2012). Similarly, structural studies of the connectome have found differences among regions in features such as degree, strength, betweenness and k-coreness (Hagmann et al., 2008; van den Heuvel and Sporns, 2013). They have also identified important structural subsystems, including communities (Hagmann et al., 2008; Betzel et al., 2014) and a densely-interconnected “rich club” backbone that ties together distant hubs (van den Heuvel et al., 2012). These findings are generally in accordance with a view of the brain as being organized along hierarchical lines, with segregated low-level processing of unimodal information taking place in the primary visual, auditory, sensory and motor cortices, higher-level representation and association of modal information taking place in the secondary cortices, and multisensory areas integrating information between distinct modalities across large-scale networks (Felleman and Essen, 1991; Yeo et al., 2011).

In this work, we propose a method to characterize the information-theoretic properties of local brain regions as well as networks of regions, here referred to as *subsystems*. Our method employs functional time series in conjunction with spatial and structural connectivity data. It uses both structure and function data in a complementary manner, as opposed to studies that assess structural or functional domains separately, or that use one domain to predict the other, such as recent work in predicting functional from structural connectivity (Honey et al., 2010; Abdelnour et al., 2014; Goñi et al., 2014).

Specifically, we looked at the amount of *functional coupling* that holds between brain regions of interest (ROI), as quantified by *predictability* or the number bits of mutual information provided about the activity of one set of regions given knowledge of the activity of another set of regions. We also looked at the amount of *integration*, or internal functional coupling within subsystems as quantified by a multivariate generalization of mutual information.

In addition, we measured the *scaling* of predictability and integration by quantifying the growth of these measures as increasingly large sets of regions are considered, an approach motivated by previous work on multi-scale integration in complex multivariate systems (Grassberger, 1986; Tononi et al., 1994; Bialek et al., 2001). In this work, subsystems were defined with respect to the structural and physical organization of the brain. In particular, three different metrics were used to define subsystems (which may overlap): *Euclidean subsystems* are maximally compact in terms of physical distance; *Connectome subsystems* are maximally compact according to shortest path distances on the connectome; and *Randomized subsystems* are maximally compact according to shortest path distances on a randomly rewired version of the Connectome.

Our methodology combines data from resting-state functional MRI [fMRI] as well as from structural deterministic fiber tractography based on DSI. We first explored the scaling of measures of predictability of individual ROIs using subsystems of different sizes chosen using Euclidean, Connectome and Randomized metrics, where larger scales correspond to subsystems containing more ROIs. Then ROIs were characterized in terms of their functional coupling to the rest of their corresponding hemisphere, as well as in terms of the Euclidean spatial range at which they maintained long-distance functional coupling. An analysis of the correlation between functional coupling and a structural measure of shortest-path efficiency between ROIs and distant neighbors was performed across different scales. Finally, we looked at scaling of multivariate measures of integration within subsystems and of functional coupling of subsystems with the rest of the hemisphere. We identified a set of Connectome-based networks whose subsystems showed a combination of high internal integration and high coupling with the rest of the hemisphere.

The rest of the paper is organized as follows. The MRI data is described in the section MRI Data and section Distance Metrics describes the three different structural metrics considered in this study, corresponding to physical proximity (Euclidean), anatomical connectivity (Connectome), and a randomized control of the Connectome (Randomized). In section Information-Theoretic Measures and Efficiency, we describe our information-theoretic measures of the predictability of ROIs and subsystems at multiple spatial scales defined by the three metrics. In section Results, we report average measures of information-theoretic scaling, variation of these measures across the cortical surface, the relationship between long-range functional and structural shortest-paths, and identify Connectome subsystems that are both internally integrated and coupled to the rest of their hemispheres. In section Discussion, we discuss the use of information theory for studying the functional organization of the brain, interpret our results in the context of the integrative functions of the cortex, and overview some methodological considerations. We finish by suggesting possible avenues for future development of our approach.

## Materials and methods

### MRI data

Forty healthy subjects underwent an MRI session on a 3T Siemens Trio scanner with a 32-channel head-coil. Magnetization Prepared Rapid Gradient Echo (MPRAGE) sequence was 1 mm in-plane resolution and 1.2 mm slice thickness, with a FOV of 256 × 240 mm, and included 160 slices. Diffusion Spectrum Imaging (DSI) sequence included 128 diffusion weighted volumes + 1 reference b0 volume, with maximum *b*-value *b* = 8000 s/mm^2^, 2.2 × 2.2 × 3.0 mm voxel size, 212 × 212 mm FOV, and 34 slices. Functional MRI Echo Planar (EPI) sequence was 3.3 mm in-plane resolution with 3.3 mm slice thickness and 0.3 mm slice gap, 212 × 193 mm FOV, 32 slices, and TR 1920 ms. DSI, resting-state fMRI and MPRAGE data were processed using the Connectome Mapping Toolkit (Daducci et al., 2012). All the processing steps were performed in the individual subject space with no spatial normalization.

Segmentation of gray and white matter was based on MPRAGE volumes. The parcellation used for all the analyses in this work divides the GM cortex into 448 ROIs (Cammoun et al., 2012); one ROI was eliminated due to signal acquisition errors, resulting in a final analysis on 447 ROIs (see Figure S1). Subcortical regions were not considered in this study. For reporting purposes, ROIs within each hemisphere were grouped into 34 larger, physically-compact *anatomical areas* corresponding to a GM anatomical atlas (Desikan et al., 2006). Figure S2 in the Supplementary Material shows the assignment of ROIs to anatomical areas.

During the resting-state fMRI acquisition, subjects were lying in the scanner with eyes open, resting but awake and cognitively alert, for a period of approximately 9 min. Functional data preprocessing included motion correction, regression of white matter, cerebrospinal fluid and movement signals, linear detrending, motion scrubbing and low-pass filtering (Fox et al., 2009; Power et al., 2012), producing a 280-sample time series for each ROI of each subject. The first four samples were removed to allow for signal stabilization, resulting in a final time series length of 276 samples per ROI per subject. Some subjects were found to have spikes in across-ROI variance of fMRI signal; maximum across-ROI variance over all time points was computed for all subjects and three subjects with outlier maximum variance were removed (outliers chosen according to Tukey's rule threshold of *upper-quartile + 1.5 × inter-quartile range*). This resulted in a final dataset containing 37 subjects (16 female, 25.3 ± 5.0 years old). The data used for the findings reported here were not processed with global signal regression. However, when global signal regression was applied, none of the reported results changed qualitatively (data not shown).

Whole brain streamline tractography was performed on reconstructed DSI data (Wedeen et al., 2008), resulting in a structural connectivity matrix where each entry reflects the number of fibers (Hagmann et al., 2008), denoted by NOF in this paper. This dataset was also assessed in two other studies (Betzel et al., 2014; Goñi et al., 2014). In this work, we did not consider inter-hemispheric connections, which pose difficulties for DWI-based deterministic fiber tractography (Gong et al., 2009) and which may be systematically underrepresented in connectomes constructed using such methods.

Finally, subjects' fMRI time series and DSI connectomes were combined into a single “pooled” subject. Though no inter-subject spatial normalization was performed, subject-wise functional time series and structural connectivity can be pooled together because they were evaluated on the basis of the same anatomical atlas registered to each individual subject space. The time series for each ROI of each subject was mean-centered, rescaled to standard deviation 1, and concatenated across subjects to yield a single time series of 276 × 37 = 10,212 samples. For the structural connectivity matrices, entries in the pooled matrices were taken to be means of the corresponding connectivity values across the individual subject connectivity matrices. Though the data for the pooled subject does not correspond to any real subject, it is more robust and generates more stable and reliable statistics, important for computing the kinds of information-theoretic measures considered in this work (see Section Methodological Considerations). For these reasons, this kind of subject-pooling is frequently performed in computational neuroscience (van den Heuvel and Sporns, 2011; Deco et al., 2013; Haimovici et al., 2013; Betzel et al., 2014; Goñi et al., 2014).

**Figure 1:**
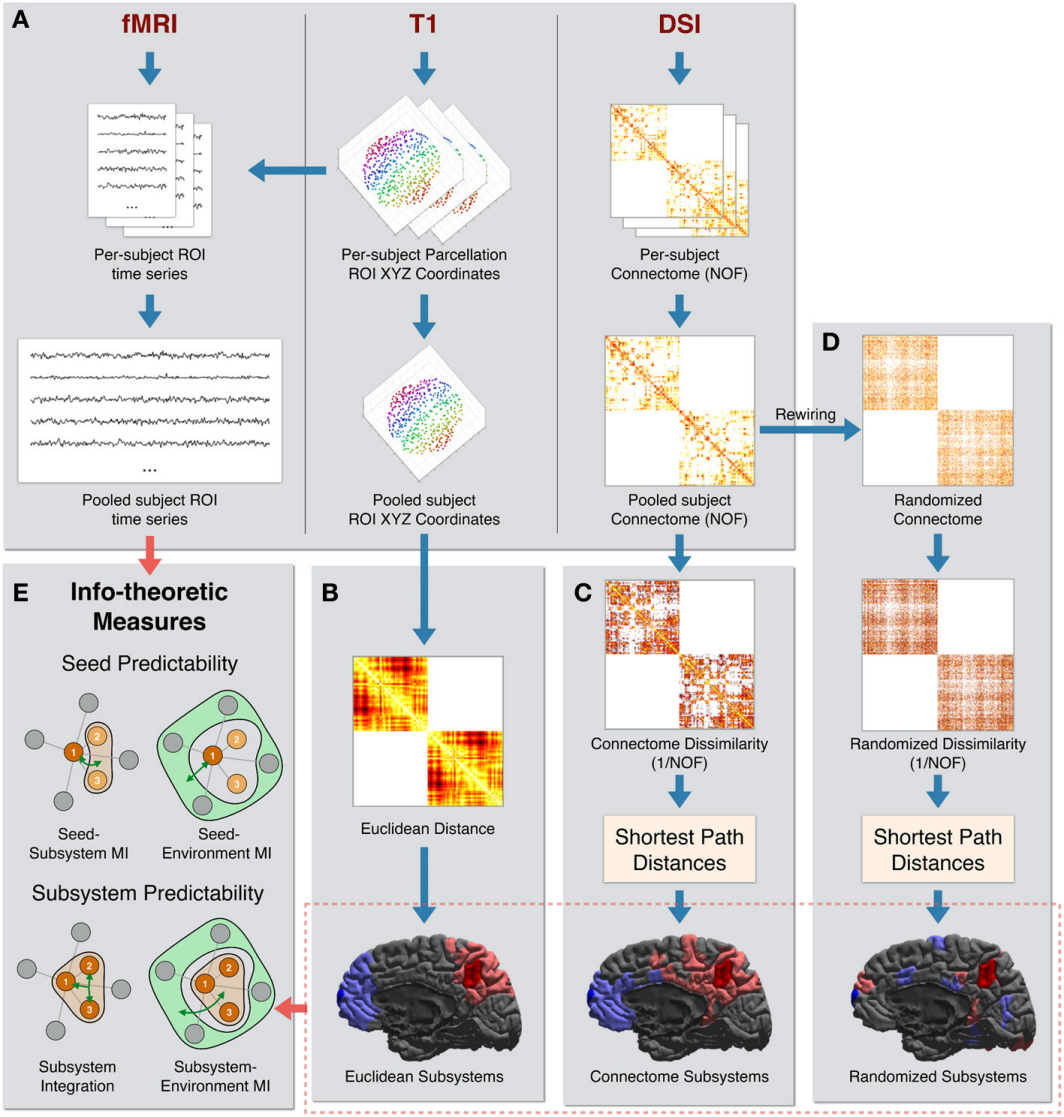
**Data processing pipeline. (A)** The steps used to create the final dataset of pooled subject ROI time series, pooled subject ROIs coordinates, and pooled connectome [using the number of fibers (NOF) measure]. **(B)** *Euclidean Distance* is computed as the physical distance between the centroids of ROI coordinates. At bottom are shown two example Euclidean sets of size 20 ROIs centered on two “seed” ROIs: one seed in the precuneus region (seed in dark red and the rest of the subsystem in light red) and one seed in the frontal pole region (seed in dark blue and the rest of the subsystem in light blue). **(C)** NOF structural connectivity matrix is transformed into a Connectome Dissimilarity matrix. Shortest paths on the dissimilarity graph are used to create the *Connectome* distance metric. Two examples of Connectome sets of 20 ROIs are shown, centered on the same two ROIs as in **(B)**. **(D)** A degree preserving rewiring is used to create a randomized structural connectivity matrix. This is transformed into a Randomized dissimilarity matrix. Shortest path distances on the corresponding dissimilarity graph are used to create the *Randomized* distance metric. Two example Randomized sets of 20 ROIs are shown, centered on the same two ROIs as in **(B)**. **(E)** The subsystems and time series are used to calculate a set of information-theoretic measures of predictability and integration.

The processing steps used to create the final dataset of pooled subject ROI time series, pooled subject ROIs coordinates, and pooled subject structural connectivity are diagrammed in Figure 1A.

### Distance metrics

As will be described in the next section, we computed measures of predictability in terms of a given ROI and its nearest neighbor ROIs. Nearest neighbors rankings were defined according to three different distance metrics: *Euclidean*, *Connectome*, and *Randomized*.

The *Euclidean* metric was defined as the Euclidean distance between the centroid coordinates of pairs of ROIs. The Euclidean neighbors of a given ROI were thus the most physically proximate ROIs. This is diagrammed in Figure 1B.

The *Connectome* metric was defined using anatomical connectivity inferred from DSI data. For the weights of structural connections linking ROIs, we used the NOF between ROIs as identified by the tractography algorithm (Hagmann et al., 2008). Since higher values of this measure indicate greater connectivity, we computed Connectome dissimilarity between anatomically connected ROIs as the inverse of the connectivity values between them (i.e., *1/NOF*). Connectome neighbors of a given ROI were other ROIs most proximate in terms of shortest-path distances on the Connectome dissimilarity graph. These processing steps are diagrammed in Figure 1C.

Finally, the *Randomized* metric was defined by first performing a degree-preserving rewiring (Maslov and Sneppen, 2002) of the Connectome graph. This rewiring method creates a randomized symmetric graph that preserves the density of the network (i.e., the number of direct connections), the degree (number of connections per ROI), and the overall distribution of NOF values. As performed in the Connectome metric, dissimilarity was computed as the inverse of the (rewired) NOF values. Analogously to the other metrics, Randomized neighbors of a given ROI were the most proximate ROIs in terms of shortest-path distances on the Randomized dissimilarity graph. These processing steps are diagrammed in Figure 1D.

As mentioned in the last section, due to possible confounding errors in inferring inter-hemispheric structural connectivity, each hemisphere was analyzed separately and only neighbors from the same hemisphere were considered for a given ROI.

We illustrate some examples of the subsystems defined according to these metrics in the bottom sections of Figures 1B–D. For each of the three metrics, two sets of 20 ROIs (a seed ROI and its 19 nearest neighbors) centered on two right-hemisphere ROIs are colored: one in the precuneus region (seed in dark red and rest of the subsystem in light red) and one in the frontal pole region (seed in dark blue and the rest of the subsystem in light blue).

As expected, sets of Euclidean neighbors (bottom of Figure 1B) are physically contiguous and compact. Connectome neighbors (bottom of Figure 1C) also tend to cluster spatially but are more distributed, with connections that span large physical distances present. In addition, according to the Connectome metric, the precuneus is close to the entire medial portion of the hemisphere (and far from more lateral regions) while the frontal pole is closer to superior and medial frontal as well as inferior temporal regions. Finally, the ROIs comprising Randomized neighbors (bottom of Figure 1D) are scattered throughout the hemisphere.

The Euclidean distance matrix as well as the Connectome and Randomized connectivity matrices are shown in Figure S3 in Supplementary Material. That figure also shows the neighbor ranks of all ROIs for all given seeds. Notably, while distances between ROIs are symmetric, ranks are not necessarily so (if one ROI is the *k*th neighbor of another ROI, the second is not necessarily the *k*th neighbor of the first).

### Information-theoretic measures and efficiency

Our information-theoretic measures of predictability were computed in terms of mutual information (MI) between different sets of ROIs. Mutual information is defined as

I(X;Y):=H(X)+H(Y)−H(X,Y)

where *H(·)* stands for the entropy function. In addition, we used a multivariate generalization of MI known as *total correlation* (Ay et al., 2006), defined as the sum of the marginal entropies for a set of random variables minus their joint entropy:

$$TC(X_{1},\ldots ,X_{k}):=\Sigma_{1\leq i\leq k}H(X_{i})-H(X_{1},\ldots ,X_{k})$$

Total correlation, *TC(·)*, quantifies the degree of multivariate correlation present in a subsystem and can be interpreted as the bits of compression gained by encoding the joint outcome of a set of random variables as opposed to encoding each variable's outcome independently. It is large when individual variables have high individual variance but are jointly correlated (for example, if all variables are copies of each other).

In this work, we considered the entropy of fMRI-recorded BOLD time-series of different brain regions. Because this data is continuous, we computed our information-theoretic measures using differential entropy (Cover and Thomas, 2012). For a random variable *X* with probability density function *p(x)*, differential entropy is defined as:

h(X)=−∫p(x)logp(x)dx

To estimate differential entropies, we used a multivariate Gaussian assumption and employed the uniformly minimum-variance unbiased estimator of multivariate Gaussian entropy (Ahmed and Gokhale, 1989). If *X* is a *k*-by-*n* matrix representing *n* samples from a *k*-dimensional multivariate Gaussian (for example, corresponding to samples of the activity of a group of *k* ROIs), this method estimates the entropy in bits of the underlying distribution as:

$$\frac{1}{\ln2}\left(\frac{k}{2}\ln e\pi +\frac{1}{2}\ln\left|XX^{\prime }\right|-\frac{1}{2}\sum_{i=1}^{k}\psi \left(\frac{n+1-i}{2}\right)\right)$$

where ψ is the digamma function. By itself, differential entropy is not guaranteed to be positive nor invariant to one-to-one coordinate transforms such as rescalings. However, mutual information and total correlation values computed using differential entropies are always positive and invariant to coordinate changes (Cover and Thomas, 2012).

We measured the information shared between sets of ROIs defined as follows. Any given ROI *i* can be considered as the target of prediction, in which case it's called the *seed*. The ROI which is the *k*th ranked neighbor of *i* is indicated by *n*_*i*_(*k*) (as described in the previous section, these can be chosen according to one of three different metrics: physical Euclidean distance, Connectome distance, or Randomized connectome distance). The seed together with its *k* − 1 most proximate neighbor ROIs comprise the *subsystem of size k* centered on *i*, indicated by *S*_*i*_(*k*). For a given seed, all of the ROIs in the same hemisphere except those that are in its subsystem of size *k* (in other words, those that are further than its *k*th neighbor, according to a given metric) belong to the *environment*, indicated by *E*_*i*_(*k*).

Given these definitions of seed, neighbor, subsystem and environment, we defined the following five measures of ROI predictability:

**Pairwise MI**, *I(i;j)*, is the MI between the activity of any two individual ROIs *i* and *j*. One particular type of Pairwise MI we consider in detail is the **Seed-Neighbor MI**, *I*(*i*; *n*_*i*_(*k*)), which is the MI between the activity of a seed ROI and the seed's *k*-ranked neighbor.

**Seed-Subsystem MI**, *I*(*i*; *S*_*i*_(*k*)\*i*), is the MI between the activity of the seed ROI *i* and the joint activity of the rest of its size-*k* subsystem. This measures how well the ROIs in a seed's size-*k* subsystem collectively predict the seed. This measure is illustrated in schematic form in Figure 1E.

**Total MI**, *I(i;V\i)*, where *V* represents the set of all the ROIs in the hemisphere, is the total amount of prediction possible about the seed using all other ROIs in the hemisphere. It is equivalent to the Seed-Subsystem MI when the subsystem corresponds to the entire hemisphere.

**Seed-Environment MI**, *I(i;E_i_(k))*, is the MI between the activity of the seed and the joint activity of the ROIs in the environment. This measure quantifies how well ROIs in the environment predict the activity of the seed ROI. This measure is illustrated in schematic form in Figure 1E.

**Euclidean Coupling Range** is the neighbor number at which Seed-Environment MI drops below a specific threshold. This quantifies the smallest spatial scale at which a seed becomes effectively functionally decoupled from the environment.

In addition, we defined two multivariate measures for measuring the integration and predictability of entire subsystems. As before, we chose *k*-sized subsystems that are centered on a given seed ROI, and we again defined the environment as the set of ROIs in a hemisphere that are not members of a given subsystem. We considered two multivariate measures:

**Subsystem Integration**, *TC(S_i_(k))*, is the total correlation of the activity of the set of ROIs in a size-*k* subsystem centered on ROI *i*. This measure is high when ROI activity is individually varied but collectively correlated, and is illustrated in schematic form in Figure 1E.

**Subsystem-Environment MI**, *I(S_i_(k);E_i_(k))*, is the MI between the joint activity of the set of ROIs in a size-*k* subsystem and the joint activity of the set of ROIs in the environment. This measure is high when there is strong functional coupling between subsystem and environment, and low when there is high functional segregation between the subsystem and the environment. This measure is illustrated in schematic form in Figure 1E.

When reporting these two subsystem predictability measures for subsystems of different sizes, we normalized them by subsystem size. This resulted in measures of **Subsystem Integration per ROI** and **Subsystem-Environment per ROI**.

Finally, we also computed correlations between ROI predictability measures and one measure reflecting long-range efficiency. **Global efficiency** (Latora and Marchiori, 2001) is the average of the inverse of all shortest-path distances between pairs of vertices. We define **long-range efficiency** for an ROI within a subsystem as the mean inverse shortest-path between the ROI and its Euclidean environment ROIs (i.e., the ROIs outside of its Euclidean subsystem). The long-range efficiency between the seed and the ROIs in the seed's Euclidean environment was computed using shortest paths defined by the three aforementioned metrics: Euclidean, Connectome, and Randomized.

## Results

As discussed in previous sections, for each ROI taken as the seed we obtained a list of neighbor ROIs ranked from most proximate to most distant according to three distance metrics (Euclidean, Connectome and Randomized). Figure S3 shows the ranks of neighbors for each seed and metric that were used to compute scaling properties of the information-theoretic measures. We looked at predictability of seed activity given the activity of neighbors, subsystems and environments of different sizes.

**Figure 2:**
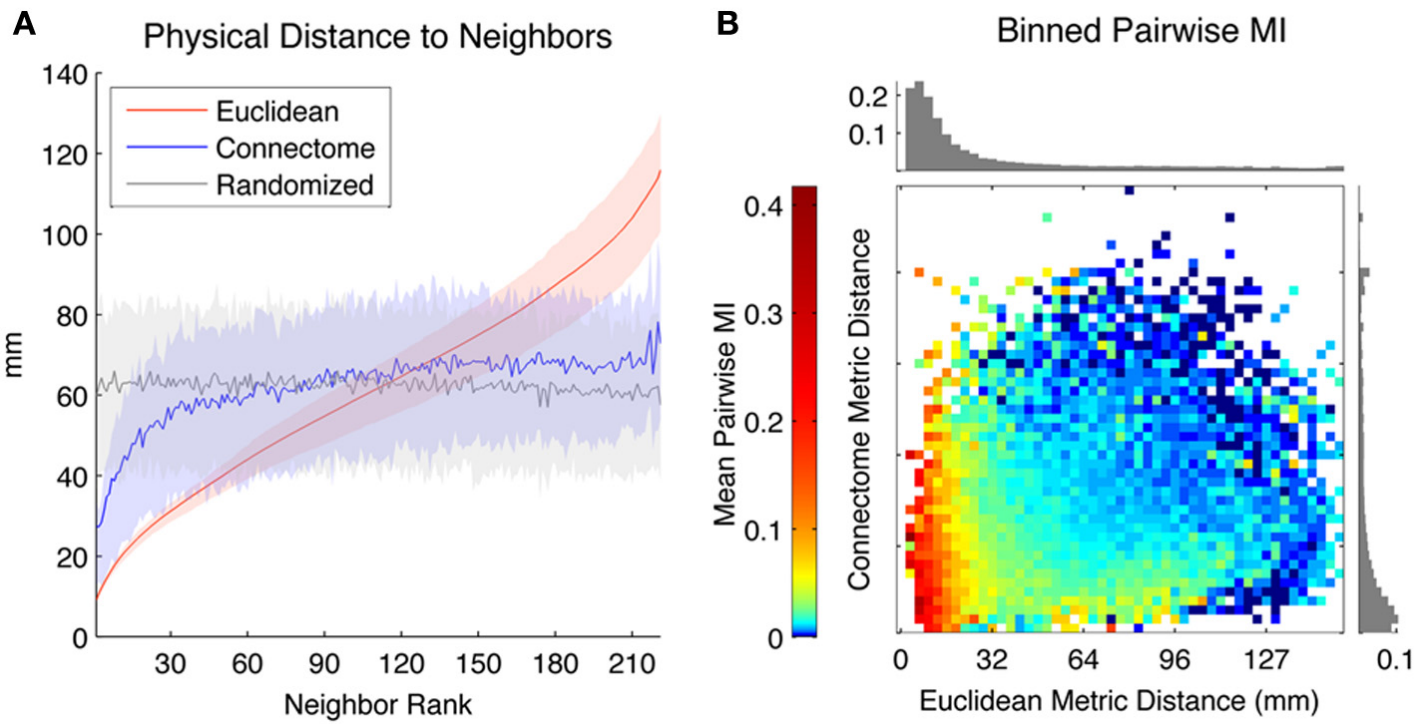
**(A)** Mean physical distance between seeds and their *k*th neighbors, where neighbors are ranked according to three metrics: Euclidean (red), Connectome (blue), and Randomized (gray) (averaged across all ROIs, with solid line representing mean physical distance and shaded areas indicating first and third quartiles). **(B)** Mean Pairwise MI between pairs of ROIs separated by different Euclidean (horizontal axis) and Connectome (vertical axis) distances. Log color scaling used to highlight differences among weakly coupled connections. Upper bar chart shows mean Pairwise MI values for pairs of ROIs separated by different Euclidean distances (irrespective of Connectome distances) while bar chart on right shows mean Pairwise MI values for pairs of ROIs separated by different Connectome distances (irrespective of Euclidean distances).

We first characterized the distance in physical space between seeds and neighbors ranked according to different metrics (Euclidean, Connectome and Randomized). In Figure 2A, the Y-axis depicts the Euclidean distance (mm) between seed ROIs and the *k*th-neighbor (X-axis) chosen according to the three metrics, averaged across all seed ROIs in both hemispheres (shaded areas reflect 1st and 3rd quartiles). The physical distance to nearby Connectome neighbors tends to be small, though highly variable across seeds and not as small as to Euclidean neighbors, which are by definition maximally proximate in physical space. Randomized neighbors display no spatial regularity, with average distance to neighbor of any rank corresponding to the expected Euclidean distance separating randomly chosen pairs of ROIs (~65 mm).

We used Pairwise MI to measure functional connectivity between pairs of ROIs as a function of their separation according to both Euclidean and Connectome distance. Euclidean and Connectome distances were divided into 50 equal-width bins and mean Pairwise MI between intra-hemispheric pairs of ROIs corresponding to each Connectome and Euclidean bin was computed. Figure 2B shows a heat map of mean Pairwise MI values within each bin (log color scaling used to better highlight differences among weakly coupled connections). The bar chart at the top of the heat map shows mean MI values for pairs of ROIs separated by different Euclidean distances (irrespective of Connectome distances), while the bar chart at the right of the heat map shows mean MI values for pairs of ROIs separated by different Connectome distances (irrespective of Euclidean distances). Overall, mean Pairwise MI tends to decrease monotonically with increasing Euclidean distance as well as with increasing Connectome distance. Pairs of ROIs that are distant according to both metrics tend to be weakly coupled (mean MI below 0.01 bits). The most strongly coupled pairs of ROIs (mean MI above 0.2 bits) are those separated by small Euclidean distances, irrespectively of Connectome distance. However, ROIs that are distant in Euclidean space but proximate on the Connectome also tend to have higher coupling (mean MI ~0.03 bits) than those that are distant in both metrics.

We next report the scaling of ROI-based predictability measures defined in section Information-Theoretic Measures and Efficiency, namely Seed-Neighbor MI, Seed-Subsystem MI and Seed-Environment MI.

**Figure 3:**
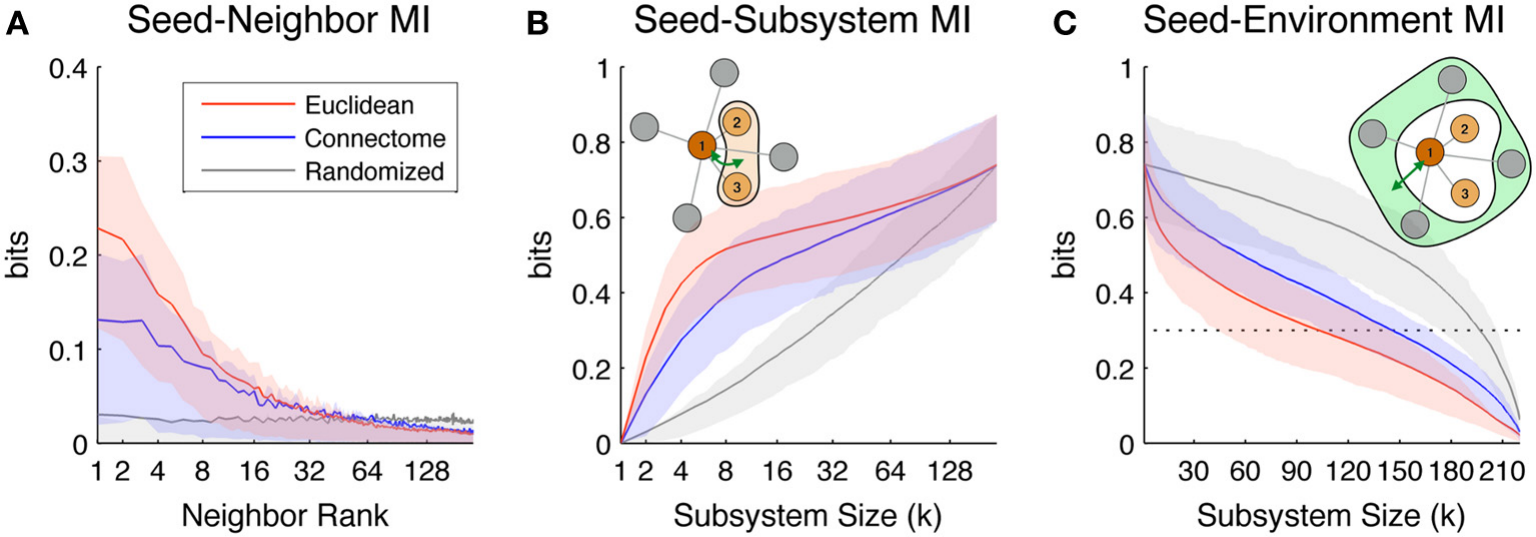
**Scaling of the information-theoretic measures of seed predictability.** Colored lines indicate mean values across all seed ROIs in both hemispheres, while shaded areas indicate values within 1st and 3rd quartile. Colors indicate values for neighbors/subsystems/environments chosen according to Euclidean (red), Connectome (blue), and Randomized (gray) distance metrics. **(A)** Average Seed-Neighbor MI between seeds and their corresponding *k*th rank neighbors chosen according to the three distance metrics. **(B)** Seed-Subsystem MI between seeds and subsystems built according to the three distance metrics. The illustration in the top left corner diagrams how this measure is computed for a given seed and subsystem of size 3. **(C)** Seed-Environment MI between seeds and environments built according to the three distance metrics. The illustration in the top right corner diagrams how this measure is computed for a given seed and subsystem size 3 (environment size 4). The horizontal dotted line indicates 0.3 bits of Seed-Environment MI, a threshold used later in our definition of Euclidean Coupling Range.

Figure 3A shows Seed-Neighbor MI between seeds and their neighbors chosen according to the three distance metrics, averaged over all ROIs in both hemispheres as seeds. ROIs that are closer in Euclidean and Connectome space have a higher MI, with closely ranked Euclidean neighbors (up to neighbor ~8) showing a higher coupling than Connectome neighbors (this reproduces the effect seen in Figure 2B, where proximate Euclidean and Connectome pairs tend to have higher Pairwise MI). As expected, Pairwise MI with Randomized neighbors displays no systematic regularity with neighbor rank. Mean Seed-Neighbor MI for Euclidean neighbors becomes most similar to the mean Seed-Neighbor MI for Randomized neighbors at approximately the 50th neighbor (for Euclidean neighbors, this corresponds to a distance of approximately 40 mm). This is the Euclidean scale at which functional correlations between pairs of physically proximate ROIs decay to baseline levels.

Figure 3B shows the scaling of Seed-Subsystem MI with increasing subsystems averaged over all ROIs in both hemispheres as seeds. The illustration in the top left corner shows in schematic form how this measure is computed (dark brown is the seed, light brown is subsystem, and green arrow is MI). Seed-Subsystem MI grows monotonically with increasingly large subsystems as more subsystem ROIs become available to predict the activity of the seed. On average, seeds have the strongest coupling to Euclidean subsystems, closely followed by Connectome subsystems. However, across the full range of subsystem sizes, there is great overlap in the distribution of Seed-Subsystem MI values for subsystems defined according to these two metrics. In contrast, Randomized subsystems display much less Seed-Subsystem MI over the entire range of subsystem sizes. The three measures converge once subsystems begin to overlap and grow toward including the entire hemisphere.

Figure 3C shows scaling of Seed-Environment MI, the multivariate coupling between the seed and the environment. The illustration in the top right corner shows in schematic form how this measure is computed (dark brown is the seed, light green is environment, and green arrow is MI). Note that since the environment is defined as the set of hemispheric ROIs outside of the subsystem, environment size decreases with increasing subsystem size. For this reason, Seed-Environment MI always decreases monotonically with increasing subsystem size, as less and less environmental ROIs are available for predicting the seed. On average, Euclidean environments tend to have less predictability about seeds than Connectome environments, indicating that sets of ROIs that are distant in space tend to be less functionally coupled to seeds than sets of ROIs distant on the Connectome. However, there is again a large overlap between Seed-Environment MI values over the range of environment sizes. Randomized environments tend to have the highest values of Seed-Environment MI. This is due to the fact that Randomized environments include more spatially- and structurally-proximate ROIs to the seeds (which tend to be highly functionally coupled; Figure 2A) than Euclidean and Connectome environments that by definition do not include ROIs that are, respectively, proximate in space or on the Connectome.

Next, we looked at how predictability of different ROIs varies across the cortical surface using two measures defined in section Information-Theoretic Measures and Efficiency: Total MI and Euclidean Coupling Range.

**Figure 4:**
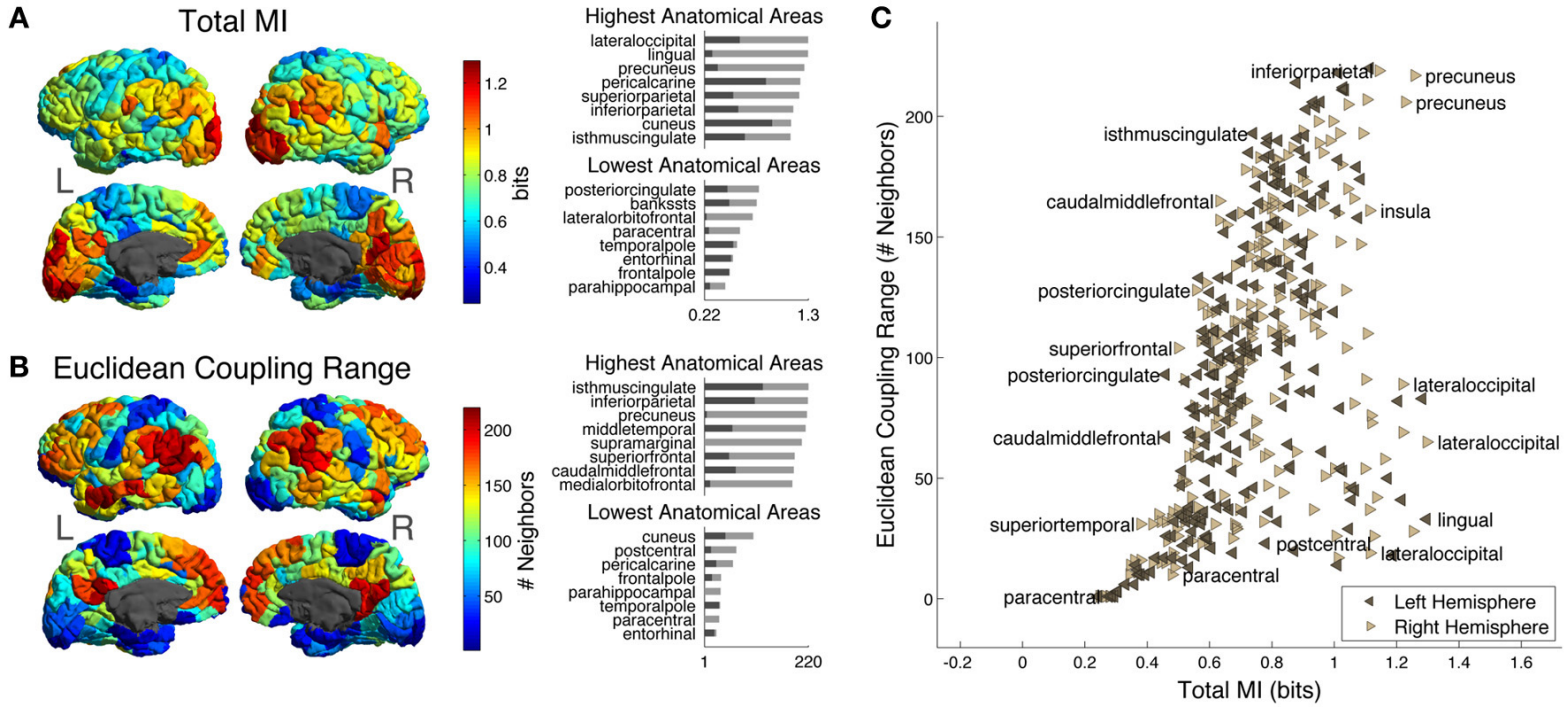
**(A)** Cortical distribution of Total MI, the total amount of predictability available about each ROI from the ROIs in the rest of the hemisphere. On the upper right are the top 8 anatomical areas arranged according to maximum Total MI of ROIs with each area (maximum Total MI of intra-area ROIs indicated by light gray bars; the minimum indicated by dark gray bars) while on the lower right are the bottom 8 anatomical areas arranged according to maximum Total MI of ROIs within each area (maximum Total MI of intra-area ROIs indicated by light gray bars; the minimum indicated by dark gray bars). **(B)** Cortical distribution of Euclidean Coupling Range, the neighbor number at which Euclidean Seed-Environment MI drops below a threshold of 0.3 bits (see text for details). On the upper right are the top 8 anatomical areas arranged according to maximum Euclidean Coupling Range of ROIs with each area [light and dark gray bars indicating maximum and minimum values as in **(A)**] while on the lower right are the bottom 8 anatomical areas arranged according to maximum Euclidean Coupling Range of ROIs with each area [light and dark gray bars indicating maximum and minimum values as in **(A)**]. **(C)** Scatter plot of Total MI vs. Euclidean Coupling Range for left and right hemisphere ROIs. A few ROIs are labeled with the names of their corresponding anatomical areas.

Figure 4A shows Total MI, or total amount of predictability available about the activity of each ROI given the rest of ROIs in the hemisphere (note that this measure does not depend on choice of distance metric). In addition, we show the distribution of Total MI in different anatomical areas. As described in section MRI Data, ROIs in each hemisphere are grouped into 34 larger-scale “anatomical areas” that correspond to the FreeSurfer parcellation (Desikan et al., 2006). The bar chart on the upper right of the cortical Total MI plot shows the top 8 anatomical areas arranged according to maximum Total MI of ROIs within each area (maximum Total MI of ROIs in each area indicated by light gray bars; the minimum indicated by dark gray bars). The areas with the 8 largest maximum Total MIs are lateral occipital, lingual, precuneus, pericalcarine, superior parietal, inferior parietal, cuneus, and isthmus cingulate. The bar chart on the lower right of the cortical Total MI plot shows the lowest 8 anatomical areas arranged according to maximum Total MI of ROIs within each area (maximum Total MI of ROIs within each area indicated by light gray bars; the minimum indicated by dark gray bars). The areas with the 8 lowest maximum Total MIs are posterior cingulate, banks of the superior temporal sulcus (bankssts), lateral orbitofrontal, paracentral, temporal pole, entorhinal, frontral pole, and parahippocampal.

We next investigated how Euclidean Coupling Range is distributed across the cortical surface, as well as its correlation with a structural measure.

Figure 4B shows the Euclidean Coupling Range of each ROI on the cortical surface. Euclidean Coupling Range, defined as the Euclidean neighbor number at which Seed-Environment MI drops below a given threshold, quantifies the maximal spatial scale at which a given amount of functional coupling with the environment is maintained. We used a threshold amount of 0.3 bits, which is the average Seed-Environment MI when half-hemisphere-sized subsystems/environments (~110 ROIs) are considered (see horizontal dotted line in Figure 3C). On the upper right are shown the top 8 anatomical areas arranged according to maximum Euclidean Coupling Range of ROIs within each area (light and dark gray bars indicating maximum and minimum values as in Figure 4A). The areas containing the 8 highest maximum Euclidean Coupling Range are isthmus cingulate, inferior parietal, precuneus, middle temporal, supramarginal, superior frontal, caudal middle frontal and medial orbitofrontal. On the lower right are shown the bottom 8 anatomical areas arranged according to maximum Euclidean Coupling Range of ROIs within each area (light and dark gray bars indicating maximum and minimum values as in Figure 4A). The areas containing the 8 lowest maximum Euclidean Coupling Range are cuneus, postcentral, pericalcarine, frontal pole, parahippocampal, temporal pole, paracentral and entorhinal.

In Figure 4C, we contrast these two measures using a scatter plot of Total MI (X-axis) vs. Euclidean Coupling Range (Y-axis) values for all left- and right-hemisphere ROIs. Several ROIs are labeled with the names of corresponding anatomical areas in order to indicate which areas tend to have high and low values of these two measures.

We then looked at the relationship between Seed-Environment MI, a measure of functional coupling, and long-range efficiency, a measure of structural connectivity, in order to assess whether structural features may be driving long-range functional coupling. Long-range efficiency (see Section Information-Theoretic Measures and Efficiency) is defined as the mean inverse shortest-path lengths between each seed ROI and the set of ROIs in its Euclidean environment (that is, its long-Euclidean-range neighbors). Seed ROIs with greater efficiency values are more proximate, according to some metric, to their long-range Euclidean neighbors than those with lower efficiency ones. To compare the accessibility of long-Euclidean-range neighbors over Connectome space vs. Euclidean and Randomized space, we computed different efficiency values corresponding to shortest-path lengths to those neighbors on the three different distance metrics.

**Figure 5:**
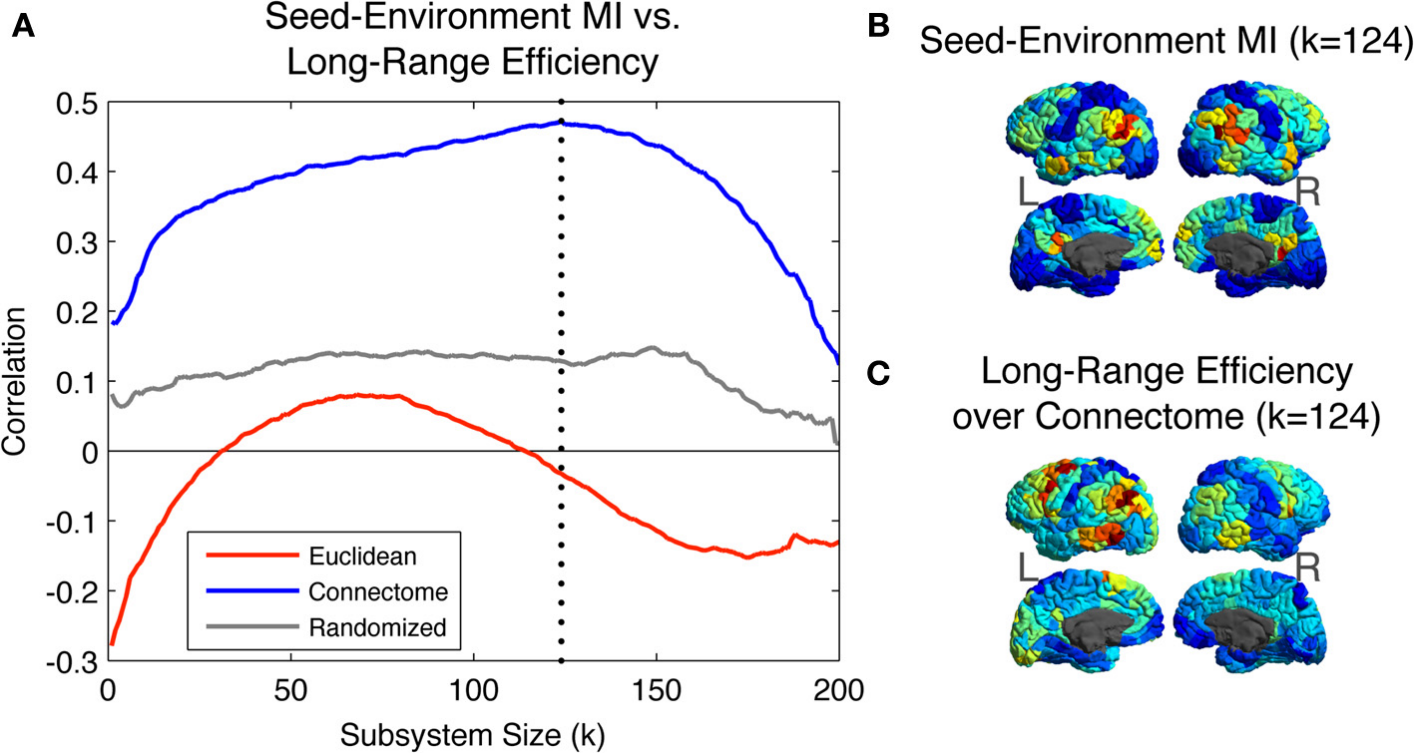
**(A)** Pearson Correlation coefficient values between Seed-Environment MIs and the long-range efficiency to environmental ROIs as increasingly distant Euclidean environments are considered. Efficiency values are computed using distances defined on the three metrics. The vertical dotted line indicates subsystem size 124, where the maximal correlation value of ~0.47 is observed, between Seed-Environment MI and Connectome efficiency. **(B)** Map of the cortical distribution of Seed-Environment MI for environments of Euclidean subsystems of size 124. **(C)** Map of the cortical distribution of Connectome efficiency values between seeds and environments of Euclidean subsystems of size 124.

Figure 5A shows the Pearson correlation values between the Seed-Environment MI and the three long-range efficiency measures as increasingly long Euclidean distances are considered (with increasing subsystem size on the X-axis, environments become increasingly small and distant). Correlations are computed separately across all seed ROIs within each hemisphere and then averaged between hemispheres. Correlations are highest between Seed-Environment MI and long-range efficiency values over the Connectome metric. They reach a peak correlation value of ~0.47 at *k* = 124 (vertical dotted line), corresponding to environments composed of ROIs located further than ~65 mm from the seed. Such a strong correlation was not observed for efficiency values computed using either of the other two metrics at any scale.

In Figure 5B, we plot for different seed ROIs the Seed-Environment MI of Euclidean environments corresponding to subsystems of size 124 (when the Connectome structural vs. functional correlation is maximal; vertical dotted line in Figure 5A). In Figure 5C, we plot the cortical distribution of the corresponding Connectome efficiency values between seed ROIs and the Euclidean environments. It can be seen that these two measures display a highly similar spatial distribution, indicating that at this scale ROIs with the highest functional coupling to long-Euclidean-range ROIs also tend to be the most efficiently connected to them over the Connectome.

So far we have looked at the predictability of individual ROIs considered as seeds. We now look at two (normalized) multivariate measures of the predictability of joint activity of entire subsystems: Subsystem Integration per ROI and Subsystem-Environment MI per ROI.

**Figure 6:**
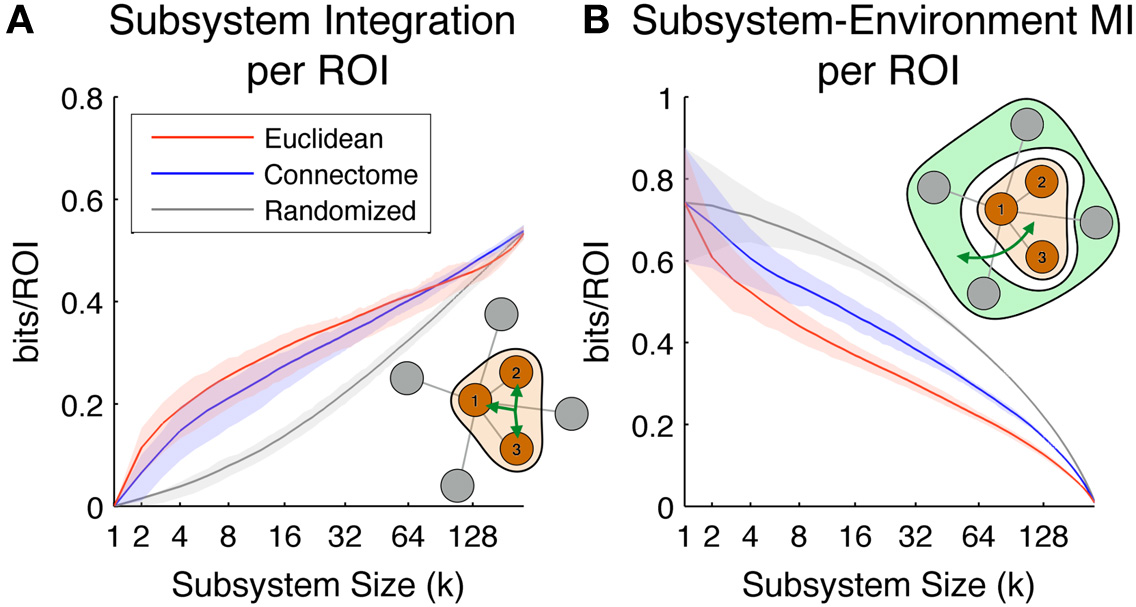
**Scaling of the subsystem predictability measures.** Colored lines indicate mean values across all subsystems, while shaded areas indicate values within 1st and 3rd quartile. Red, blue, and gray colors correspond to subsystems chosen according to Euclidean, Connectome and Randomized metrics respectively. **(A)** Subsystem integration per ROI, showing total correlation in the joint activity of ROIs in subsystems of different sizes. The illustration in the lower right corner diagrams how this measure is computed for a given subsystem of size 3. **(B)** Subsystem-Environment MI, showing functional coupling between subsystems and environments for different sizes. The illustration in the top right corner diagrams how this measure is computed for a given subsystem of size 3 and its environment.

Figure 6A shows the Subsystem Integration per ROI, which quantifies the amount of total correlation of subsystem activity (divided by subsystem size for normalization purposes). The diagram in the lower right of the figure shows in schematic form how this measure is computed (brown is the subsystem, and the three-pointed green arrow is total correlation). On average, the most integrated subsystems up to size ~90 ROIs are those defined according to the Euclidean metric (size-90 Euclidean subsystems have a radius of ~55 mm), while subsystems defined according to the Connectome are on average the most integrated for larger subsystem sizes. As expected, subsystems selected according to the Randomized metric, which are neither spatially co-located nor densely structurally interconnected, display a much lower level of multivariate integration.

Figure 6B shows Subsystem-Environment MI per ROI, a measure of the mutual information between subsystems and their environments (divided by subsystem size for normalization purposes). The diagram in the upper right of the figure shows in schematic form how this measure is computed (brown is subsystem, light green is environment, and the green arrow is MI). On average this measure is lowest for Euclidean subsystems, indicating that these are more functionally segregated from the rest of the hemisphere than subsystems defined according to the other metrics. Interestingly, Connectome subsystems are nearly as segregated as Euclidean ones at small scales (up to subsystem size ~10), but at larger scales they are more functionally coupled to the rest of the hemisphere. Randomized subsystems have the highest Subsystem-Environment MI for all the scales, since they are composed of groups of ROIs scattered through the brain and their boundaries are spanned by many pairs of ROIs separated by short Euclidean and Connectome distances (which tend to have high functional connectivity).

**Figure 7:**
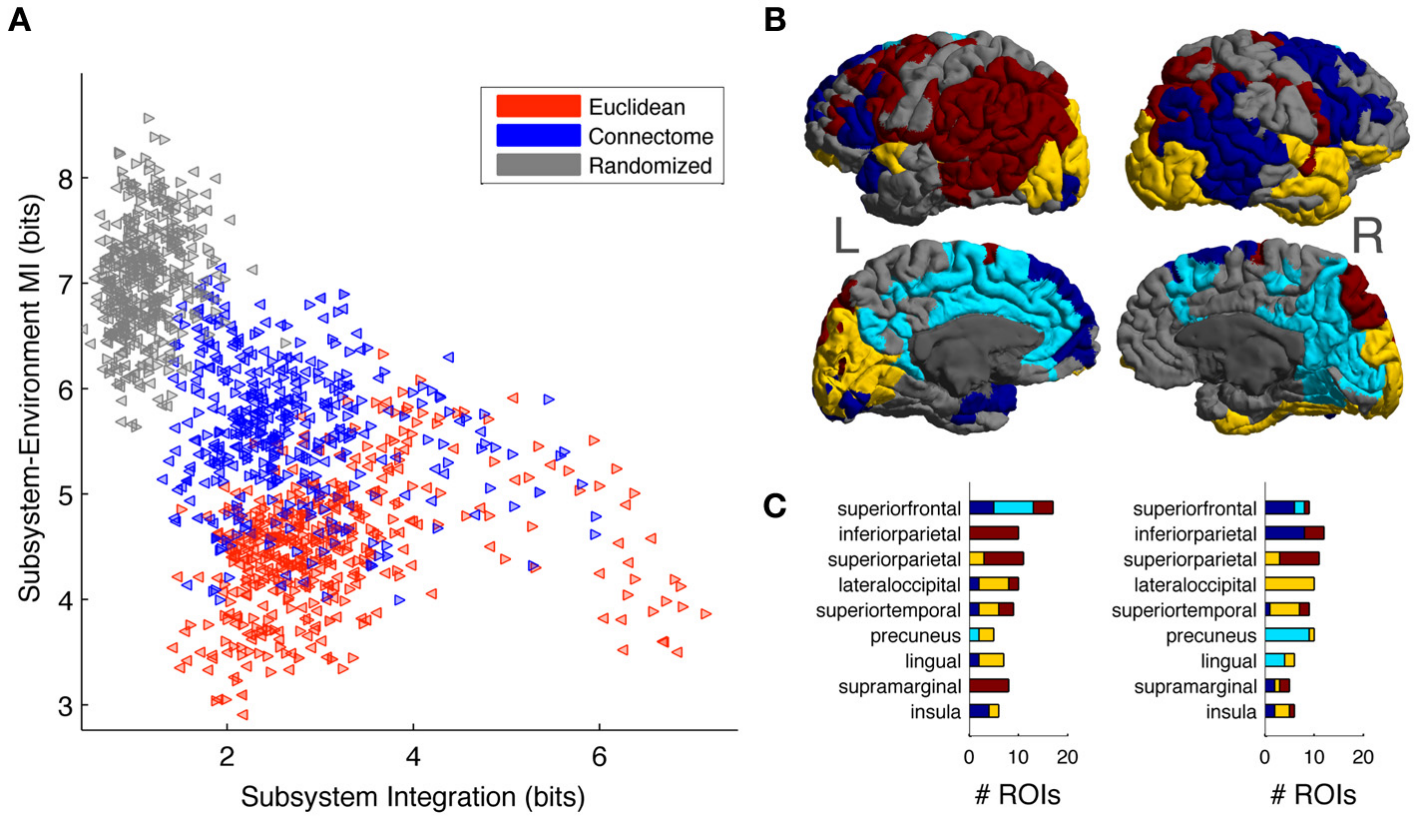
**(A)** Scatter plot of Subsystem Integration vs. Subsystem-Environment MI for subsystems of size 11, with red, blue and gray colors correspond to subsystems chosen according to Euclidean, Connectome and Randomized metrics respectively. Left-hemisphere subsystems are indicated with left-pointing triangles and right-hemisphere subsystems are indicated with right-pointing triangles. **(B)** Connectome subsystems in the upper 50 percentile of both Subsystem Integration and Subsystem-Environment MI were chosen and allowed us to identify four minimally overlapping “subsystem communities” in the left and right hemispheres. ROIs are colored according to community membership (color arbitrary); gray ROIs are those that did not belong to any high-Subsystem-Integration, high-Subsystem-Environment MI subsystem. **(C)** The distribution of subsystem communities across anatomical areas. Bar chart shows the number of ROIs from each community that are contained in different anatomical areas for the top 9 represented anatomical areas. Bar chart colors correspond to the colors used on the cortical map.

Overall, Figure 6 shows that Connectome subsystems exhibit both high Subsystem-Environment MI and high Subsystem Integration. We explored this finding in more depth in the following figure. First, we selected all Connectome subsystems of size of 11, corresponding to a volume of approximately 5% of each hemisphere (as seen in Figure 6A, at this size Connectome subsystems are on average nearly as integrated as Euclidean subsystems but, as Figure 6B shows, contain much information about their environments). Figure 7A shows the scatter plot of Subsystem Integration (X-Axis) vs. Subsystem-Environment MI (Y-Axis) for size-11 subsystems defined according to Euclidean, Connectome and Randomized metrics. Randomized Subsystems (gray) tend to cluster in regions of the scatter plot characterized by high Subsystem-Environment MI (lack of segregation from environment) and low Subsystem Integration (lack of internal integration). Euclidean Subsystems (red) tend to occupy regions of the scatter plot characterized by low Subsystem-Environment MI (high segregation from environment) and high Subsystem Integration (high internal integration). Connectome Subsystems (blue), however, occupy intermediate regions of the scatter plot, demonstrating significant amounts of both Subsystem-Environment MI (thus not being functionally segregated from the rest of the hemisphere) while also having significant Subsystem Integration (thus also having internal integration).

We investigated which specific Connectome subsystems maximize both Subsystem Integration and Subsystem-Environment MI. First, Connectome subsystems that were in the upper 50 percentile of both measures in each hemisphere were selected. Next, because these subsystems overlapped (contained some of the same ROIs; see Figure S4A), we clustered them into a smaller number of minimally-overlapping “subsystem communities.” To do so, for each hemisphere we computed a subsystem-by-subsystem Overlap Matrix whose entries measured the proportion of ROIs shared between each pair of subsystems (see Figure S4B, S4C for the left and right hemisphere Overlap Matrices). A community-detection algorithm (Blondel et al., 2008) was run on this matrix to provide a partition of the subsystems into communities.

The community-detection algorithm identified four communities in the left hemisphere and another four in the right hemisphere. Figure 7B shows cortical surface of the left and right hemisphere, with each ROI colored according to its membership in a subsystem community (colors arbitrary but selected so that communities that have similar spatial distributions in both hemispheres have the same color). ROIs that belong to more than one selected subsystem were assigned to their most frequent community. Gray colored ROIs are those that were not part of any subsystem that was in the top 50 percentiles according to the two MI measurements.

Finally, we looked at how the subsystem communities obtained were distributed across anatomical areas. Anatomical areas were ranked in terms of their participation in the subsystems maximizing Subsystem Integration and Subsystem-Environment MI. Figure 7C lists the top 9 anatomical areas: superior frontal, inferior parietal, superior parietal, lateral occipital, superior temporal, precuneus, lingual, supramarginal, and insula. The stacked bar charts indicate, for both hemispheres, the number of ROIs from each subsystem community that are contained in each anatomical area, with bar chart colors corresponding to the colors used on the cortical map. We discuss the distribution of these subsystem communities across anatomical areas in more detail in the section Subsystem Predictability and Integration vs. Segregation Trade-Off.

## Discussion

In this work, we characterized brain regions and networks in terms of their information-theoretic measures by using both functional and structural information in a complementary manner. The measures presented here quantify the amount of functional coupling between sets of ROIs as well as integration within sets of ROIs. Sets of ROIs form subsystems which are selected according to three different possible distance metrics: Euclidean (reflecting the physical spatial embedding of brain regions), Connectome (reflecting the anatomical structural connectivity of the brain), and Randomized (a comparison condition based on a rewired version of the Connectome graph; see Section Distance Metrics). We also investigated the scaling of these measures, in the sense of their growth as larger subsystems are considered.

In section Information-Theoretic Measures for Studying the Organization of the Brain, we discuss the use of information-theoretic measures for characterizing the brain and the need for such measures to account for the brain's spatial and topological embedding. In section Scaling of Information-Theoretic Measures, we discuss the scaling of our measures as brain subsystems of different sizes are considered, their distribution across the cortical surface, and their relation to long-range efficiency. In section Subsystem Predictability and Integration vs. Segregation Trade-Off, we discuss the fact that Connectome subsystems tend to be highly internally integrated while also being coupled to the rest of the brain, and that the subsystems that optimize this trade-off cluster into communities that resemble previously identified resting state networks. In section Methodological Considerations we discuss some important methodological considerations and assumptions involved in this work. In the last section Future Directions, we suggest some possible directions for further work.

### Information-theoretic measures for studying the organization of the brain

As mentioned in the Introduction, much recent research has been devoted to characterizing the structural and functional roles of different brain regions and networks. Many of these characterizations have identified certain regions as structural and functional hubs, decomposed the brain into weakly-coupled modules and networks, and investigated the role of large-scale integrative backbones.

Information theory provides a natural language for talking about systemic aspects of the organization of functional brain activity, including the presence of quasi-independent modular subsystems and the integrative properties of functional hubs and networks. Several information-theoretic measures for studying brain organization have been proposed in the literature. One measure of particular interest is TSE complexity (Tononi et al., 1994), which is based on the idea that low-level processing is performed in localized, segregated brain regions that operate in parallel and interconnect along hierarchical lines, while high-level association and integration is performed in large-scale distributed networks (Felleman and Essen, 1991; Yeo et al., 2011). TSE complexity quantifies this notion by looking at the scaling of total correlation as increasingly large subsystems are considered. The degree to which the total correlation of large-scale regions (having many components) exceeds that of small-scale regions (containing few components) is a quantitative signature of integration at large scales.

Importantly, the activity of the brain unfolds across physical space and structural connectivity networks. For this reason, it can be expected to qualitatively follow Tobler's *first law of geography*: “Everything is related to everything else, but near things are more related than distant things” (Tobler, 1970). In fact, as previously reported (Salvador et al., 2005; Honey et al., 2009; Power et al., 2013) and as also shown here, functional interactions are stronger between spatially proximate regions. TSE complexity, however, considers integration at a given scale by looking at all possible subsets of component of a given size. Thus, while it represents a promising step toward an information-theoretic treatment of large-scale integration, it disregards spatial and connectivity information and the fact that the organization of functional activity is often dominated by physically localized interactions.

In this work, we looked at the scaling of information-theoretic measures across both physical space and the Connectome, and compared it to scaling over the Randomized metric (which, like TSE, disregards actual spatial and topological organization). As we will discuss, we found that the Randomized metric poorly represents the functional organization of our brain data, and this weakness may also be expected of TSE. In fact, underlying spatial and connectome structure must be taken into account in order to properly quantify the amount of large-scale integration in the brain. In addition, our methodology, which captures systematic relationships between the size and the strength of functional constraints in spatially-compact subsystems, allows us to compute localized information-theoretic measures of scaling. This allows for the characterization of the variation of integration and predictability across the cortical surface.

### Scaling of information-theoretic measures

We measured the amount of functional coupling between each ROI (the “seed”) and the set of most proximate neighbors (the local “subsystem”) as well as the set of most distant neighbors (the distant “environment”). We also computed how the strength of functional coupling scales as increasing numbers of neighbors are chosen according to one of the three different distance metrics—Euclidean, Connectome, or Randomized.

As discussed in Section Information-Theoretic Measures for Studying the Organization of the Brain, functional activity organized according to an underlying metric will display stronger functional coupling between nearby locations vs. more distant ones. Thus, information-theoretic measures computed on sets of ROIs chosen according to a more “representative” space are expected to give rise to higher values of Seed-Subsystem MI and Seed-Neighbor MI for close neighbors (i.e., more integration within local regions) as well as lower values of Seed-Environment MI (i.e., more segregation between local regions and the rest of the system).

According to these criteria, both Euclidean and Connectome metrics better represent the functional organization of resting state activity than the Randomized metric (Figure 3). On average, for small scales, the Euclidean metric captures more strong functional couplings than does the Connectome metric, as shown by higher values of Seed-Neighbor MI (Figure 3A) and Seed-Subsystem MI (Figure 3B) measures for Euclidean vs. Connectome subsystems. Generally, for the range of scales considered, ROIs chosen according to the Connectome metric display an amount of functional coupling with neighbors between those chosen according to the Euclidean metric on one hand and Randomized on the other. We discuss some possible reasons for the intermediate role played by Connectome subsystems below.

The strength of functional coupling between seeds and Euclidean subsystems (Figure 3B), as well as the fact that the environments chosen in terms of distant Euclidean neighbors display the least functional coupling (Figure 3C), demonstrates that resting-state brain data is highly spatial, in that it exhibits strong correlations over small Euclidean scales (some reasons for this are discussed below in section Methodological Considerations). However, while short-Euclidean range interactions are strong, the brain also integrates information globally and exhibits functional coupling over large spatial scales. Because Seed-Environment MI quantifies the functional coupling between seed ROIs and remote locations, we defined Euclidean Coupling Range as the number of Euclidean neighbors at which the Seed-Environment MI drops below a threshold of 0.3 bits, and looked at the distribution of this measure across the cortical surface (Figure 4).

This measure was found to have a highly heterogeneous distribution across the brain. Low values of Euclidean Coupling Range—indicating that only short-scale correlations present—are found in unimodal sensorimotor cortices, including locations corresponding to V1, motor areas in the precentral gyrus, somatosensory areas in postcentral gyrus, paracentral areas corresponding to the supplementary motor area, and superior temporal areas corresponding to auditory cortex. On the other hand, locations in the brain having high Euclidean Coupling Range—indicating the presence of long-range functional couplings—include recognized high-level hub areas (van den Heuvel and Sporns, 2013), such as the precuneus, inferior parietal, superior frontal gyrus, anterior cingulate, temporoparietal junction and ventral frontal cortex. In addition, regions thought to have functional roles at intermediate levels of the cortical hierarchy, such as higher-order visual and auditory cortices as well as somatosensory association cortices, tend to display intermediate values of Euclidean Coupling Range.

Importantly, variation in Euclidean Coupling Range arises due to variation in the range of spatial coupling of different ROIs and is not simply due to differences in their inherent level of predictability. We compared Euclidean Coupling Range with Total MI, a measure of mutual information between each ROI and the rest of the ROIs in its hemisphere. Total MI does not rely on any underlying metric and quantifies the inherent predictability of different regions. This measure also displayed a heterogeneous distribution across the brain, indicating that during resting-state some ROIs are much more predictable than others. Regions with the highest predictability included large areas of the occipital lobe, primarily corresponding to the primary and higher-order visual cortices, as well as some regions of the parietal lobe such as the inferior parietal lobule. Notably, many regions high in Euclidean Coupling Range—such as those in the frontal lobe—did not have exceptionally high Total MI, nor did many regions with high Total MI—such as visual cortices—have high Euclidean Coupling Range (Figure 4C). Thus, Euclidean Coupling Range is a continuous measure that separates regions having spatial segregation (low values) from those having spatial integration (high values) and identifies functional hubs at multiple scales of the cortical hierarchy. Our results are in agreement with previous research showing a connection between functional hubs and long-spatial-range functional coupling (Sepulcre et al., 2010).

We also evaluated whether functional coupling between spatially distant regions may be driven by long-rage efficiency. Hence we correlated Seed-Environment MI and long-range efficiency (over the three metrics) between ROIs and their Euclidean environments for a wide range of scales. For most scales, long-range efficiency over the Connectome was positively correlated with the Seed-Environment MI, while correlations were much smaller with long-range efficiency over Euclidean and Randomized metrics. Thus, the presence of Connectome shortest-paths between the seed and spatially distant ROIs was the best predictors of strong functional coupling between them, reflecting a possible fingerprint of structural connections in driving functional coupling over large spatial scales.

### Subsystem predictability and integration vs. segregation trade-off

Our information-theoretic approach measured not only the predictability of seed ROIs, but also the multivariate predictability of sets of ROIs in subsystems (Figure 6). We investigated two complementary measures: Subsystem-Environment MI and Subsystem Integration. On average Connectome subsystems displayed nearly as much Subsystem Integration as Euclidean subsystems up to subsystem size 90, and more integration for larger sizes. Across many scales, Euclidean subsystems located in the occipital lobe (corresponding to the visual cortices) displayed the highest amounts of integration (these subsystems, for example, are the cluster of points with very high integration shown in the scatter plot of Figure 7A). On the other hand, in comparison to Euclidean subsystems, Connectome subsystems had a higher Subsystem-Environment MI, indicating that they were less functionally segregated from the rest of the hemisphere. Randomized subsystems were much less internally integrated and much less segregated from their environments than either Euclidean or Connectome subsystems.

At first glance, subsystems with high functional integration are also expected to display high functional segregation. The fact that Connectome subsystems have relatively high values of both Subsystem Integration and Subsystem-Environment MI suggests that they may balance a trade-off between two important information-processing functions: accessing information from large areas of the brain and integrating it efficiently across a network of hub regions (Zamora-López et al., 2010). We investigated this question by looking at particular values of Subsystem Integration and Subsystem-Environment MI for subsystems of size of 11 (~5% of one hemisphere) (Figure 7A). We chose Connectome subsystems with high values on both Subsystem-Integration and Subsystem-Environment MI and found that they are distributed into four minimally-overlapping subsystem communities (Figure 7B). Interestingly, these communities can be interpreted in terms of neural anatomy as well as in terms of previous work on functional resting state networks. The yellow communities in the left and right hemispheres occupy areas corresponding to primary and secondary visual and auditory cortices, the light blue communities roughly correspond to locations in the default mode network, while the dark red and blue communities contain regions reported to be part of the ventral attention, dorsal attention, and fronto-parietal control resting state networks (Yeo et al., 2011). The anatomical regions (Figure 7C) most represented in the light blue, dark blue and dark red communities are known to include many functional hub regions, such as superior frontal gyrus, inferior and superior parietal lobules, supramarginal gyrus and insula. Interestingly, in both hemispheres, the superior frontal gyrus included ROIs corresponding to all three of these communities, suggesting that it may be a location where these separate high-level integrative networks intersect.

Overall, this shows that our multivariate information-theoretic measures provide useful characterization of integration and coupling in subsystems. Furthermore, we found that they identify regions that display large values of integration and coupling, some of which are similar to previously reported resting-state networks.

### Methodological considerations

The Randomized metric was used as a control for comparison and was not expected to correspond closely to the functional organization. On the other hand, the fact that nearby Connectome neighbors exhibited increased functional coupling (Figure 2B) suggests that connections captured in the DSI data do correspond to actual anatomical connections that drive neural interactions and produce correlations in the multivariate BOLD signal. However, proximity in physical space, as captured by the Euclidean metric, corresponded to even higher correlations. The strong correlations between physical neighbors is driven in part by the overlap between structural and Euclidean neighbors, in that anatomical connections are enriched in spatially proximate regions (Honey et al., 2009). However, other causes may also be responsible, including undetected connections (such as local cortico-cortical connectivity and subcortical-mediated circuitry) as well as spatial smoothing due to BOLD-signal blurring due to vasculature effects, head motion artifacts, and MRI preprocessing (Honey et al., 2009; Power et al., 2012, 2013).

The framework proposed here looks at the scaling of information-theoretic measures. It is not tied to any particular way of estimating information-theoretic measures from empirical data and can be applied both to continuous and discrete data. However, as discussed in section Information-Theoretic Measures and Efficiency, for practical purposes in this work we assumed that the activity of ROIs was distributed as a multivariate Gaussian. Due to the Gaussian assumption, the covariance matrix of each hemisphere's multivariate fMRI time series served as a sufficient statistic for all of our measures of predictability and integration. In addition to the Gaussian assumption, we also combined the time series from all 37 subjects into a single “pooled” subject possessing ~10,000 time points (see Section MRI Data). Because of the subject pooling, enough time points were acquired to get a reasonable estimate of the entries in this covariance matrix (defined by nearly ~20,000 parameters). We thus could estimate information-theoretic measures for high dimensional spaces, such as for the entropies of the joint activity of the ~220 ROIs present in each hemisphere.

Computing predictability using the Gaussian assumption is equivalent to predicting the activity of seed ROIs and subsystems by linear regression. The drawback of using the covariance matrix for estimating information-theoretic quantities is that it disregards non-linear interactions between ROI activities, as well as interactions of higher-order than pairwise. Though it has been suggested that bivariate fMRI time series are sufficiently Gaussian to not warrant the estimation of non-linear effects in functional connectivity (Hlinka et al., 2011), there are a number of estimators that could be used that do take into account such effects, such as for example nearest-neighbor estimators (Kozachenko and Leonenko, 1987; Singh et al., 2003; Kraskov et al., 2004; Lizier et al., 2011). However, these estimators require of a large number of samples for reliable estimates and in our case gave unstable entropy estimates (data not shown). Overall, questions about the importance of non-linear and higher-order interactions in describing the functional organization of the brain present great interest for future investigation using our framework.

For similar reasons, it was not feasible to accurately estimate our multivariate information-theoretic measures using individual subjects' time series, which included only 276 samples per ROI per subject. Our method of subject pooling, which was performed for reasons of statistical estimation, is defensible because resting state functional activity is known to be fairly similar across healthy subjects (Damoiseaux et al., 2006). In addition, structural connectivity is also similar enough across healthy subjects so that connectome pooling can be used to reduce the effect of DSI-tractography false negatives (i.e., undetected fibers) (Hagmann et al., 2008; de Reus and van den Heuvel, 2013). However, this approach prohibits us from investigating questions of inter-subject variation in information-theoretic measures as well as their relation to individual-subject structural measures. Questions of inter-subject variability of information-theoretic measures also present great interest for future investigation, which may become feasible given the availability of datasets containing longer fMRI time series.

### Future directions

As mentioned, with longer recordings it may be possible to investigate the role of non-linear coupling and higher-order interactions in the functional organization of the human brain, as well as the inter-subject variability of information-theoretic measures. In addition, it may be possible to apply these measures in a time-dependent manner in order to look for evidence of dynamic re-organization of the integrative properties of different regions. Another interesting avenue of development would be to apply our methodology to task-dependent datasets in order to test differences in information-theoretic measures exhibited under different cognitive loads and tasks. Finally, recent work on using entropy measures for diagnostic purposes (Mäki-Marttunen et al., 2013) suggests that the kinds of measures developed here may hold promise as possible sources of diagnostic markers.

Generally, the idea of using information-theory to study the functional organization of the brain draws connections to fields of statistical learning, coding theory, statistical physics, complex systems and other fields that are playing a central part in modern computational and systems neuroscience. It may also be relevant to recent ideas regarding the criticality of brain functional activity. Criticality is a concept closely tied to long-range scaling of correlations, and it has been shown in models that information-theoretic measures of integration (Erb and Ay, 2004; Feldman et al., 2008; DeDeo and Krakauer, 2012) are maximized at critical parameter values. As we have argued, however, properly measuring the scaling of integration should take into account underlying topologies on which system constraints are organized. This suggests that our approach may be useful for investigating the hypothesis that brain is poised at or nearby a critical state (Haimovici et al., 2013; Marinazzo et al., 2013).

## Funding

Artemy Kolchinsky received summer research support from the Indiana University School of Informatics through the NSF IGERT program in Brain-Body-Environment Systems. Martijn P. van den Heuvel was supported by Netherlands Organization for Scientific Research Grant VENI-451-12-001 and a fellowship of the Brain Center Rudolf Magnus. Alessandra Griffa was supported by the Swiss National Science Foundation (Schweizerische Nationalfonds Grant 320030-130090). Luis M. Rocha was supported by Intelligence Advanced Research Projects Activity (Open Source Indicators) and Indiana University Collaborative Research Grant. Olaf Sporns and Joaquín Goñi were supported by the J. S. McDonnell Foundation.

### Conflict of interest statement

The authors declare that the research was conducted in the absence of any commercial or financial relationships that could be construed as a potential conflict of interest.
