# Supplementary material for: Multi-scale integration and predictability in resting state brain activity
Source: Front Neuroinform. 2014 Jul 24;8:66. doi: 10.3389/fninf.2014.00066 (PMC4109611; doi:10.3389/fninf.2014.00066)
Supplement: Supplementary file 1 [file Presentation1.PDF]

Supplementary Material

Multi-scale Integration and Predictability in Resting State Brain Activity

Artemy Kolchinsky[1,2], Martijn P. van den Heuvel[3], Alessandra Griffa[4,5], Patric Hagmann[4,5], Luis M. Rocha[1,2], O. Sporns[6] and Joaquín Goñi[6*]

[1]*Department of Informatics, School of Informatics and Computing, Indiana University, Bloomington, IN, USA*
[2]*Instituto Gulbenkian de Ciência, Oeiras, Portugal*
[3]*Department of Psychiatry, Rudolf Magnus Institute of Neuroscience, University Medical Center Utrecht, Netherlands*
[4]*Signal Processing Laboratory 5, Ecole Polytechnique Fédérale de Lausanne, Lausanne, Switzerland*
[5]*Department of Radiology, Lausanne University Hospital (CHUV) and University of Lausanne (UNIL), Lausanne, Switzerland*
[6]*Department of Psychological and Brain Sciences, Indiana University, Bloomington, IN, USA*

*** Correspondence: Joaquín Goñi, Psychological and Brain Sciences, Indiana University, 1101 E 10th St, Bloomington, IN, 47405, USA. jgonicor@indiana.edu**

1. Supplementary Figures and Tables

1.1. Supplementary Figures

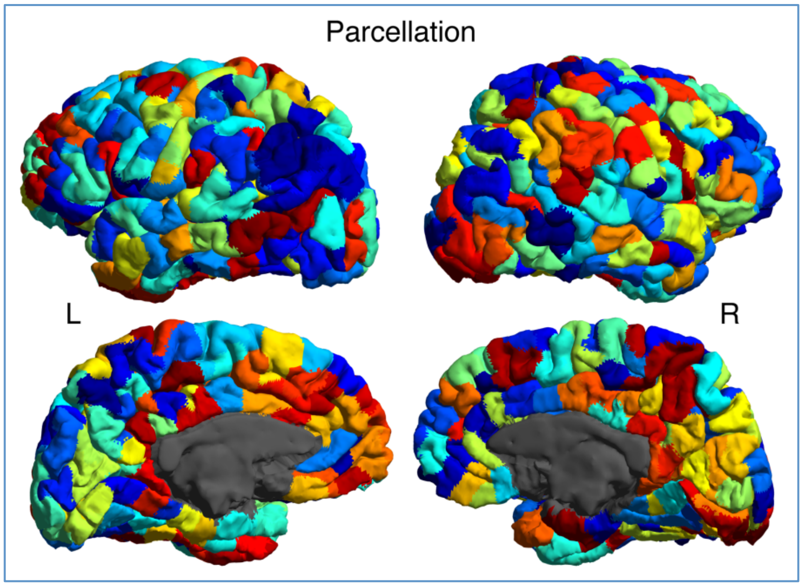

Supplementary Figure 1. Parcellation map. Colors indicate different regions of interest (ROIs). 447 cortical ROIs (225 in the left hemisphere) are present in the parcellation used for this study.

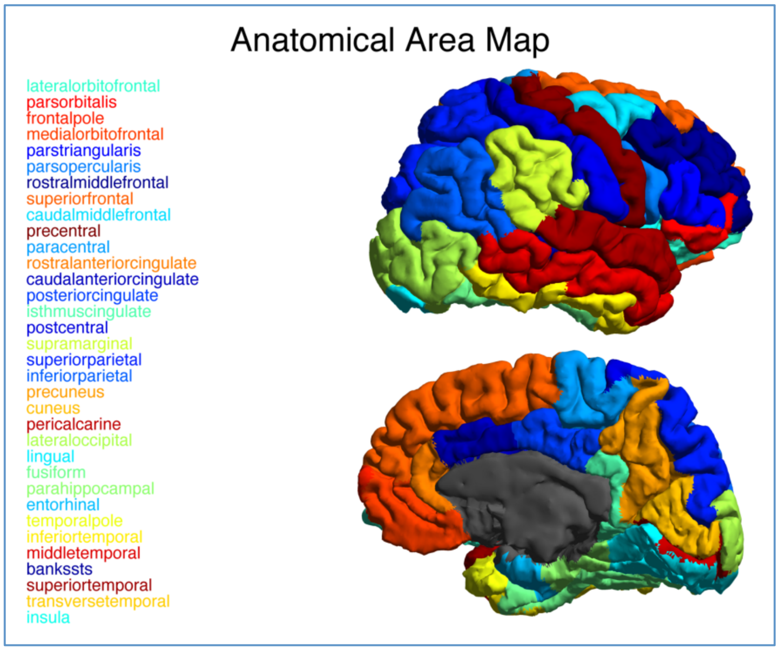

Supplementary Figure 2. Parcellation into anatomical areas. In some cases, we report averages for larger-scale annotated groups of ROIs, which we call anatomical areas. There are 34 cortical anatomical areas or regions in each hemisphere. Here, we use arbitrary colors to show the assignment of ROIs to anatomical areas.

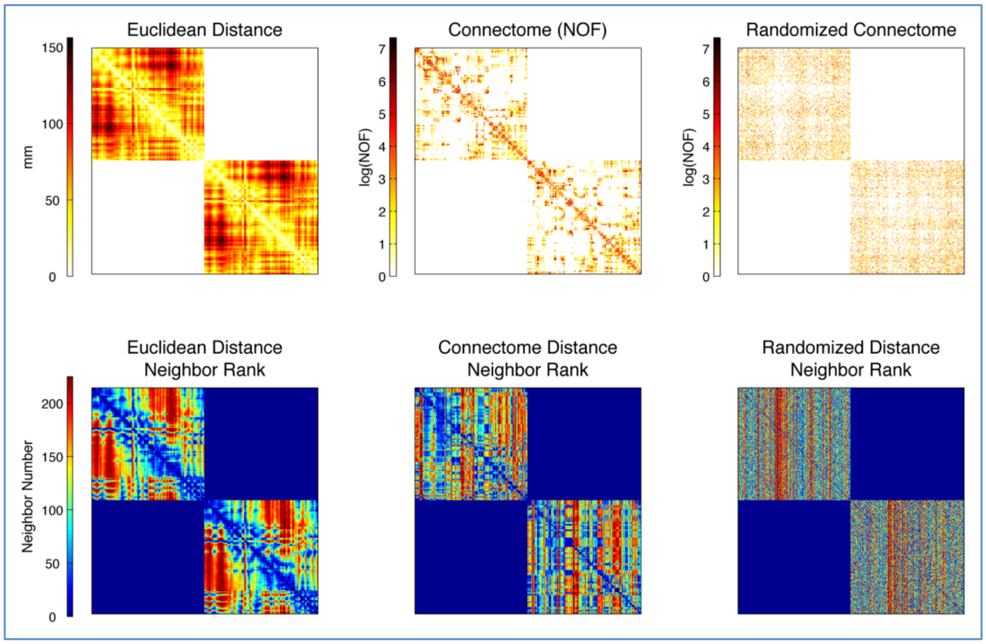

Supplementary Figure 3. Euclidean, Connectome and Randomized metrics. Top row: The distance matrix corresponding to the Euclidean metric, the Number of Fibers (NOF) Connectome matrix, and the Randomized Connectome Matrix (see the main text). The block structure represents the connections in the right hemisphere (top left) and left hemisphere (bottom right), with all inter-hemispheric connections eliminated. **Bottom row:** Euclidean, Connectome, and Randomized Subsystems are built using nearest neighbors ranked according to shortest-path metrics on the

corresponding dissimilarity matrices. Here, each row corresponds to one starting seed ROI and each column indicates the neighbor rank of the ROI corresponding to the column. Note that while the distance and connectivity matrices (top row) are symmetric, the ranking of neighbors according to each metric (bottom row) is not necessarily so. In other words, ROI j being the kth neighbor of ROI i does not necessarily imply that ROI i is the kth neighbor of ROI j.

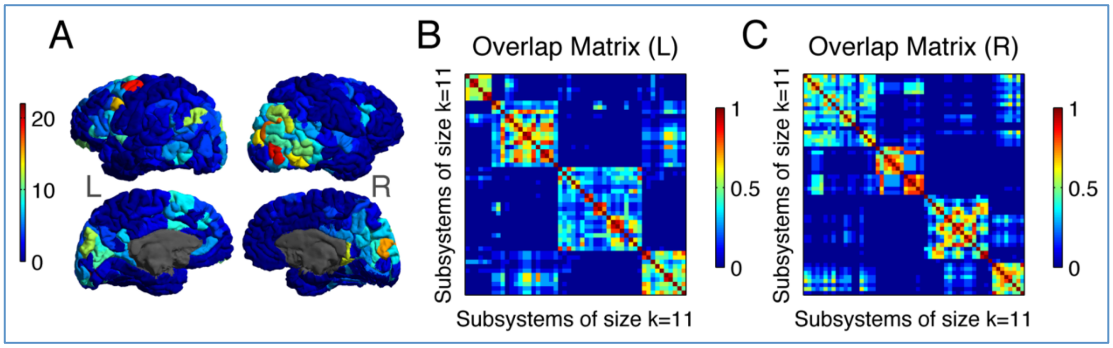

Supplementary Figure 4. High-Integration / High-Coupling Subsystems. (A) Participation of each ROI in subsystems with high Subsystem-Integration and high Subsystem-Environment MI coupling. Colors indicate in how many such subsystems each ROI participates. (B) Left Hemisphere overlap between High-Integration/High-Coupling subsystems. Colors indicate proportion of ROIs shared between each pair of subsystems. Matrix ordered according to the optimal modularity solution (see main text, 4 modules found). (C) Right Hemisphere overlap between High-Integration/High-Coupling subsystems. Colors indicate proportion of ROIs shared between each pair of subsystems. Matrix ordered according to the optimal modularity solution (see main text, 4 modules found).
